# Fatty acids and pregnancy-induced hypertension: a Mendelian randomization study

**DOI:** 10.1186/s12944-023-01889-x

**Published:** 2023-08-16

**Authors:** Zixuan Ma, Wenkai He

**Affiliations:** 1 Guangzhou Medical University, Guangzhou, China; 2 Department of Cardiology, Guangzhou Institute of Cardiovascular Disease Guangdong Key Laboratory of Vascular Diseases, The Second Affiliated Hospital of Guangzhou Medical University, Guangzhou, China

**Keywords:** Pregnancy-induced hypertension, Mendelian Randomization, Fatty acids, Causality

## Abstract

**Background:**

It is well known that pregnancy-induced hypertension (PIH) contributes significantly to the mortality rates of both mothers and babies during pregnancy. The relationship between fatty acids (FAs) and PIH remains debatable, with the causality between the two yet to be definitively established.

**Methods:**

Two-sample univariable and multivariable Mendelian Randomization (MR) analyses were executed, based on pooled data from Genome-Wide Association Studies (GWAS), to investigate any causal impact of FAs on PIH. A suite of methods was employed to assess causality, including inverse variance weighting (IVW), weighted median, MR Egger, simple mode, and weighted mode. Subsequently, the data underwent a sensitivity analysis (using Leave-One-Out analysis), a heterogeneity test (with MR-PRESSO and Cochran’s Q test), as well as a multiple validity test (using MR-Egger regression). In multivariable analyses, fatty acids were first grouped to observe the effect of individual FAs on PIH. Subsequently, factors such as diabetes, high blood pressure, and body mass index (BMI) were incorporated into a multivariable examination of the impact of each FA on PIH. During this process, the IVW, weighted median, MR-Lasso, and MR-Egger methods were employed.

**Results:**

A systematic investigation was conducted into the causal impact of each FA on PIH. The findings indicated that Polyunsaturated Fatty Acids (PUFA), Omega3, the ratio of Omega6 to Omega3, and Docosahexaenoic Acid (DHA) have a causal relationship with PIH. Increases in PUFA, Omega3, and DHA could potentially reduce the risk of PIH, while an increase in the Omega6/Omega3 ratio could heighten the risk. The impacts of other FAs (including Total Fatty Acids, Monounsaturated Fatty Acids (MUFA), Saturated Fatty Acids (SFA), and Omega 6) on PIH were not substantiated by the MR analysis. In the univariate leave-one-out analysis, rs174564 was identified in PUFA, Omega3, and DHA as having a significant role. The tests with MR-Egger and MR-PRESSO found that the results were not influenced by pleiotropy and heterogeneity. After adjusting for BMI, Diabetes Mellitus, and pre-existing hypertension in the multivariable analysis, the results mirrored those obtained univariable.

**Conclusion:**

The research implies that elevated levels of circulating PUFA, DHA, and Omega3 may serve as a protective mechanism against PIH, while higher Omega6/Omega3 ratios could potentially increase the risk of PIH. These findings may inform clinical strategies for PIH prevention.

**Supplementary Information:**

The online version contains supplementary material available at 10.1186/s12944-023-01889-x.

## Introduction

Hypertensive disorders of pregnancy (HDP) encompass both pregnancy-induced hypertension (PIH) and chronic hypertension coexisting with pregnancy [1]. The database referenced in this study focuses on conditions stemming from PIH, including pre-eclampsia and eclampsia [2, 3].

PIH is a leading contributor to maternal and neonatal mortality, with its prevalence rising annually. After delivery, pregnant mothers with PIH are more likely to suffer from hypertensive encephalopathy, renal failure, stroke, and left ventricular failure post-partum. For the neonate, it may result in severe adverse outcomes such as intrauterine growth restriction, premature delivery, fetal distress, low birth weight, inadequate oxygen supply, and fetal death. These complications can induce abnormalities across various infant systems and have long-term health impacts [4–6].

The current study explores fatty acids’ contentious and unclear causality on PIH. Some research has reported comparatively low levels of n-3 fatty acids in hypertensive pregnant women [7, 8]. Several clinical trials have also suggested the potential role of n-3 fatty acid supplementation in reducing the risk of HDP and pre-eclampsia, although these results have been inconsistent [9–12]. A recent 2022 meta-analysis found no beneficial effect of n-3 PUFA in reducing the risk of PIH and pre-eclampsia [13], while a 2020 meta-analysis indicated that n-3 fatty acid supplementation could decrease the risk of pre-eclampsia [14]. These discrepancies and controversies among studies may be due to the influence of confounding factors, leading to inaccurate results. To date, no study has definitively established the causal relationship between fatty acids and PIH, underscoring the need to investigate the influence of fatty acids on PIH using an alternative approach.

An insight into causal effects from exposure to outcome can be obtained through Mendelian randomization (MR) [15]. It is an analytical method that utilizes genetic instrumental variables (IVs) obtained from non-experimental data. The value of MR methods is that they allow for the mitigation of residual confounding, the avoidance of reverse causality, and the handling of scenarios in which exposure variables are complex to quantify or expensive [15]. Rather than being confounded by an outcome’s confounding factors, an instrumental variable is a quantifiable entity associated with an exposure of interest. By indirectly influencing the outcome via the exposure under investigation, it contributes to the proposed causal pathway. Genetic variation is not the only instrumental variable; changes in national policies, geographical location or environment, and educational level can also serve as instrumental variables [15]. In MR studies, genetic variation is a valid instrument. Single nucleotide polymorphisms (SNPs) are commonly used as IVs in genome-wide association studies (GWASs) since a random assignment of parental alleles is made to the offspring, similar to random grouping in randomized controlled trials. Therefore, MR is often considered a natural RCT. The model is unaffected by traditional factors and respects temporal order (cause precedes effect), preventing reverse causality [15, 16]. Accordingly, this research aims to conduct a two-sample MR analysis using pooled data from large-scale, open-access GWASs to investigate the causal relationships between different FAs and PIH.

## Materials and methods

### Mendelian randomization assumptions

Mendelian Randomization (MR) operates under three critical assumptions [15]. Assumption 1: The genetic variants serving as instrumental variables (IVs) must exhibit a strong association with the exposures regarding lipid-related traits in this study. Assumption 2: These IVs must not be associated with any potential confounding variables. Assumption 3: Genetic variations must only be able to influence outcomes through risks, excluding any alternative pathways. As part of our study, the logic of two-sample MR was used to determine whether fatty acids (FAs) may have a causal effect on the outcome of PIH.

**Fig. 1:**
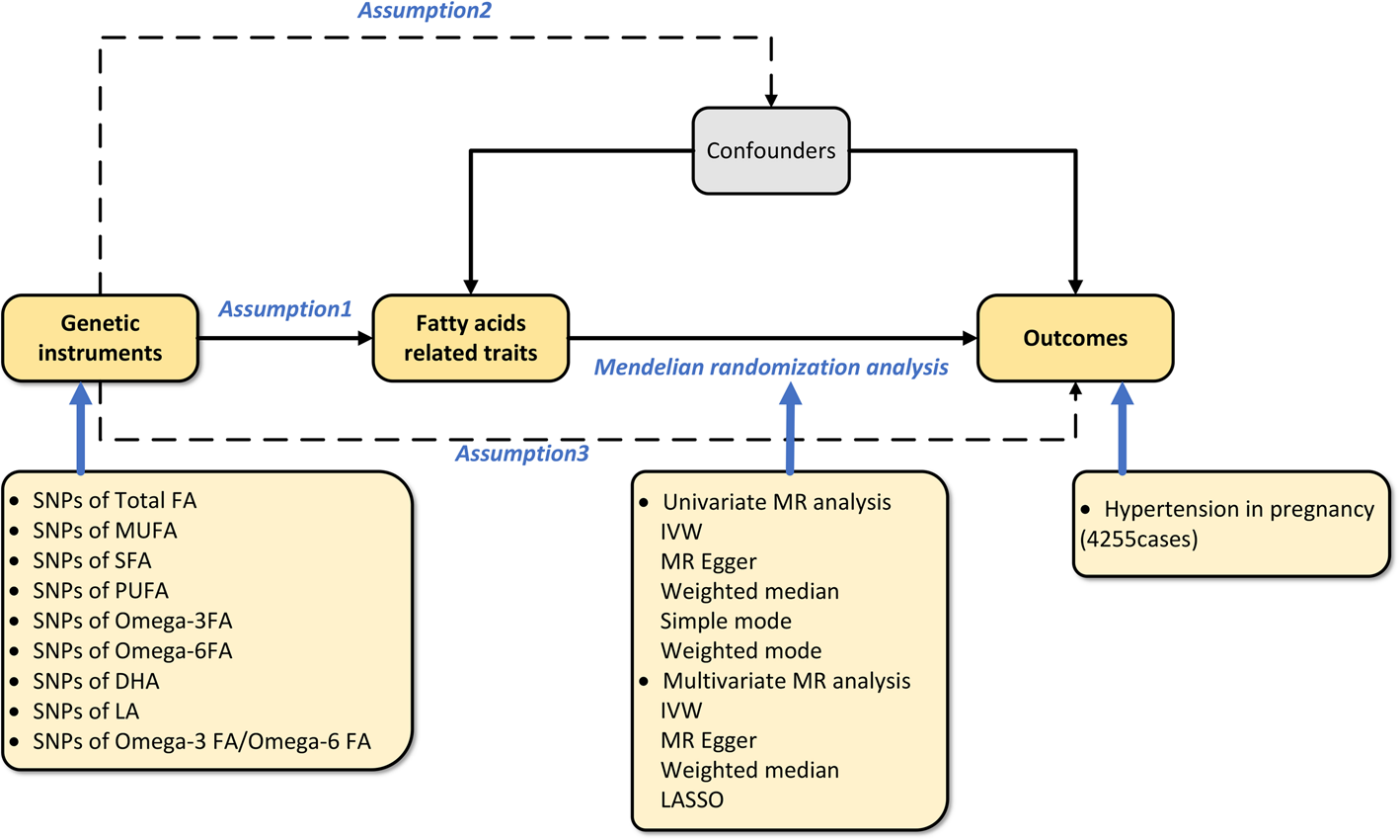
Flowchart of the Mendelian Randomization (MR) study. Based on assumption 1, fatty acids (FAs) are significantly related to genetic instrumental variables (IVs). Assumption 2 proposes that these IVs do not influence outcomes via confounding variables. Assumption 3 asserts that the genetic IVs do not directly impact the outcome of pregnancy-induced hypertension (PIH) but exert influence only through indirect exposure

Univariable MR analyses were employed to scrutinize the association between individual FAs and PIH outcomes. In contrast, multivariable MR analyses were utilized to discern the independent impact of FAs on PIH outcomes, accounting for other contributing factors or adjusting for risk factors associated with PIH presence. Figure 1 presents the experimental study design.

### Data source and open-GWAS statistics

UK Biobank (UKB) is a renowned biomedical data repository that gathers genetic and clinical data from half a million UK participants [17]. Similarly, the FinnGen study, unique in its approach, intertwines genomic data with digital healthcare records from individuals aged 18 and above, residing in Finland [18]. Its database boasts a rich compilation of prospective epidemiological cohorts, disease-based cohorts, and hospital biobank samples [18, 19].

Contrastingly, The Integrative Epidemiology Unit (IEU) OpenGWAS project primarily encompasses publicly accessible datasets, providing a valuable resource for diverse analyses, including MR analysis.

Previous research has categorized fatty acids (FAs) into four primary types: total FA, saturated FA, polyunsaturated FA, and monounsaturated FA. Further, this study analyses two specific polyunsaturated FAs—Omega-3 and Omega-6 which are predominantly comprised of DHA and LA, respectively. The individual analysis of these FAs was necessitated by the persisting ambiguities in their causal relationships, as evidenced by the contradictions in numerous studies.

The FA Genome-Wide Association Studies (GWAS) were sourced from the UKB, incorporating data from 114,999 European- ancestry participants. The PIH GWAS was derived from FinnGen, which encompasses 4,255 PIH patients and 114,795 controls, all of European ancestry, thereby eliminating potential ethnic bias. Given the distinct databases, we can assume no population overlap. All data were procured via the IEU OpenGWAS platform (IEU OpenGWAS project (mrcieu.ac.uk). Comprehensive details of this study are presented in Table 1.

**Table 1:**
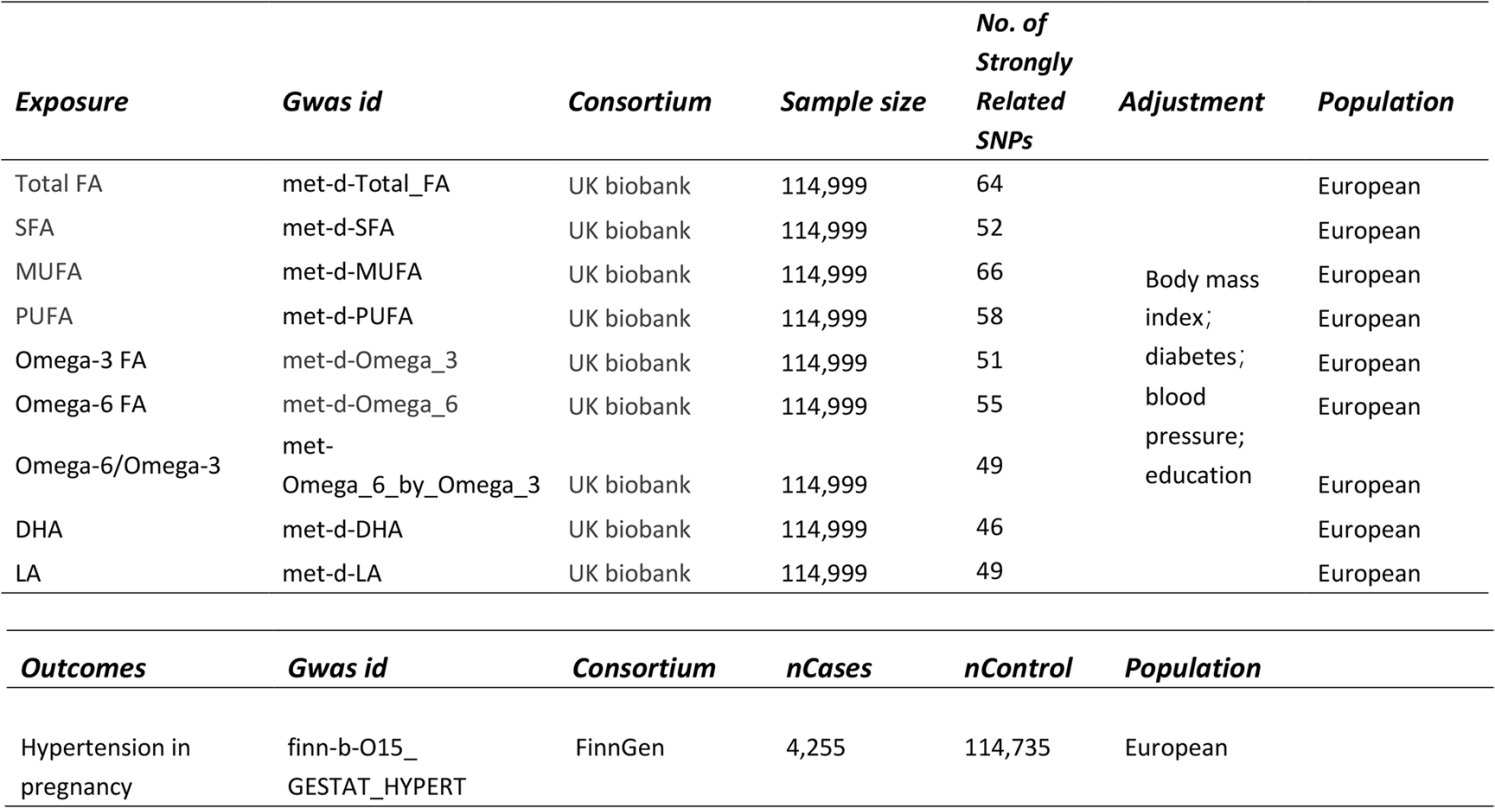
The data information used in this article

### SNP selection

To ensure adequate instrumental variables (IVs) for fatty acids (FAs) that would maintain statistical power, single nucleotide polymorphisms (SNPs) significantly associated with FAs (p < 5 × 10^-8) were selected as IVs. Subsequent steps involved removing linkage disequilibrium (LD)-linked genes, and applying thresholds of r^2 < 0.001 and Kb > 10,000. Our next step was to extract the integrative values of the selected IVs from the "PIH outcome" dataset. The second MR assumption was addressed by utilizing the PhenoScanner V2 database (http://www.phenoscanner.medschl.cam.ac.uk/) to remove SNPs associated with PIH outcomes [20]. Palindromic SNPs are defined as those with alleles corresponding to paired nucleotides in the DNA molecule, whereas a moderate allele frequency is characterized by an allele frequency within the range of 0.01 to 0.30. In MR analysis, it’s necessary to remove palindromic SNPs [21]. These may play a role in FA-PIH relationships, including obesity, diabetes, and hypertension [22].

### MR analysis

In the univariable MR analysis, five MR methods were employed to explore the causal effects of fatty acids (FAs) on PIH outcomes. The inverse variance weighted (IVW) method was the primary MR analysis technique, with MR Egger, weighted median, simple mode, and weighted mode methods serving as supplementary tools [23]. The results of MR analyses were presented as *P*-values, odds ratios (ORs), and 95% confidence intervals (CIs). MR Egger and MR-PRESSO methods were used for heterogeneity and pleiotropy testing; a *P*-value > 0.05 signified the absence of heterogeneity and pleiotropy. MR-PRESSO was also utilized for the detection and correction of potential multiplicative outliers [23]. The Cochran’s *Q* statistic was used to analyze heterogeneity through the MR Egger and IVW methods, with *P* > 0.05 indicating the absence of heterogeneity. A "leave-one-out" sensitivity analysis was performed to verify that the causal effect of FA on PIH outcome was not affected by a single SNP. Any SNP significantly affecting the outcome was earmarked for reanalysis and treated cautiously.

In addition, a multivariable MR analysis was conducted to assess the independent causal effects of relevant FA traits on PIH outcomes. This method allows for balancing the effects of similarly associated or different risk factor classes on the outcomes, thus producing more objective results. Here, the multivariable IVW and lasso analysis methods were applied to remove multiple covariate SNPs in the presence of various exposures, enhancing the accuracy of the results. Similar factors, such as fatty acids, were selected for multivariable analysis: M1 consisted of DHA, MUFA, LA, and SFA, M2 comprised MUFA, Omega3, Omega6, and SFA; M3 included MUFA, PUFA, SFA, and M5 contained Omega3 and Omega6. This classification aimed to minimize repeated or correlated SNP effects. Secondly, a multivariable MR of fatty acids was conducted with confounding factors strongly correlated with PIH, including BMI, diabetes, and high blood pressure, intending to reduce the effect of these confounding factors on fatty acid SNPs and thereby minimizing bias on the outcome.

### Statistical analysis

The R packages ‘devtools,’ 'TwoSampleMR,' 'MR-PRESSO,' 'MendelianRandomization', and ‘MVMR’ were utilized for data analysis. The Bonferroni method was employed for multiple test corrections in univariable analysis. A *P-*value < 0.0055 (0.05/9 exposures) was considered statistically significant for causality. Given the conservative nature of the Bonferroni correction [24, 25], a *P*-value between 0.05 and 0.0055 was also regarded as potentially causal. Furthermore, it is proposed that the Bonferroni method is particularly apt for multivariable MR analysis, given that the P-value correction hinges on the count of null hypotheses.

## Results

### Univariable MR

Post-running MR-PRESSO, outliers were removed, and the tests were repeated until no outliers were left. In this study, all MR analyses were performed post-outlier elimination. As illustrated in Table 2, genetically predicted fatty acids (Total FA, SFA, MUFA, Omega-6FA, LA) were not associated with PIH, as indicated by *P*-values exceeding 0.05, calculated using the IVW method and others. Nevertheless, genetically predicted PUFA [IVW, OR (95%CI): 0.80(0.66–0.98), *P* = 0.029], Omega-3FA [IVW, OR (95%CI): 0.82(0.73–0.92), *P* = 0.001], Omega6/Omega3 ratio [IVW, OR (95%CI): 1.15 (1.05–1.30), *P* = 0.018], and DHA [IVW, OR (95%CI): 0.86(0.75–0.97), *P* = 0.018] were associated with PIH. Notably, the *P*-value for Omega-3FA was below 0.0055, suggesting a robust statistical significance for causality. The remaining three *P*-values fell within the range of 0.0055 and 0.05. Given the conservative nature of the Bonferroni Correction method, these three variables could also be deemed to exhibit a causal relationship. It was found that only the Omega6/Omega3 ratio had an odds ratio (OR) greater than 1, marking it as a risk factor for PIH. This ratio might provide more clinical utility.

**Table 2:**
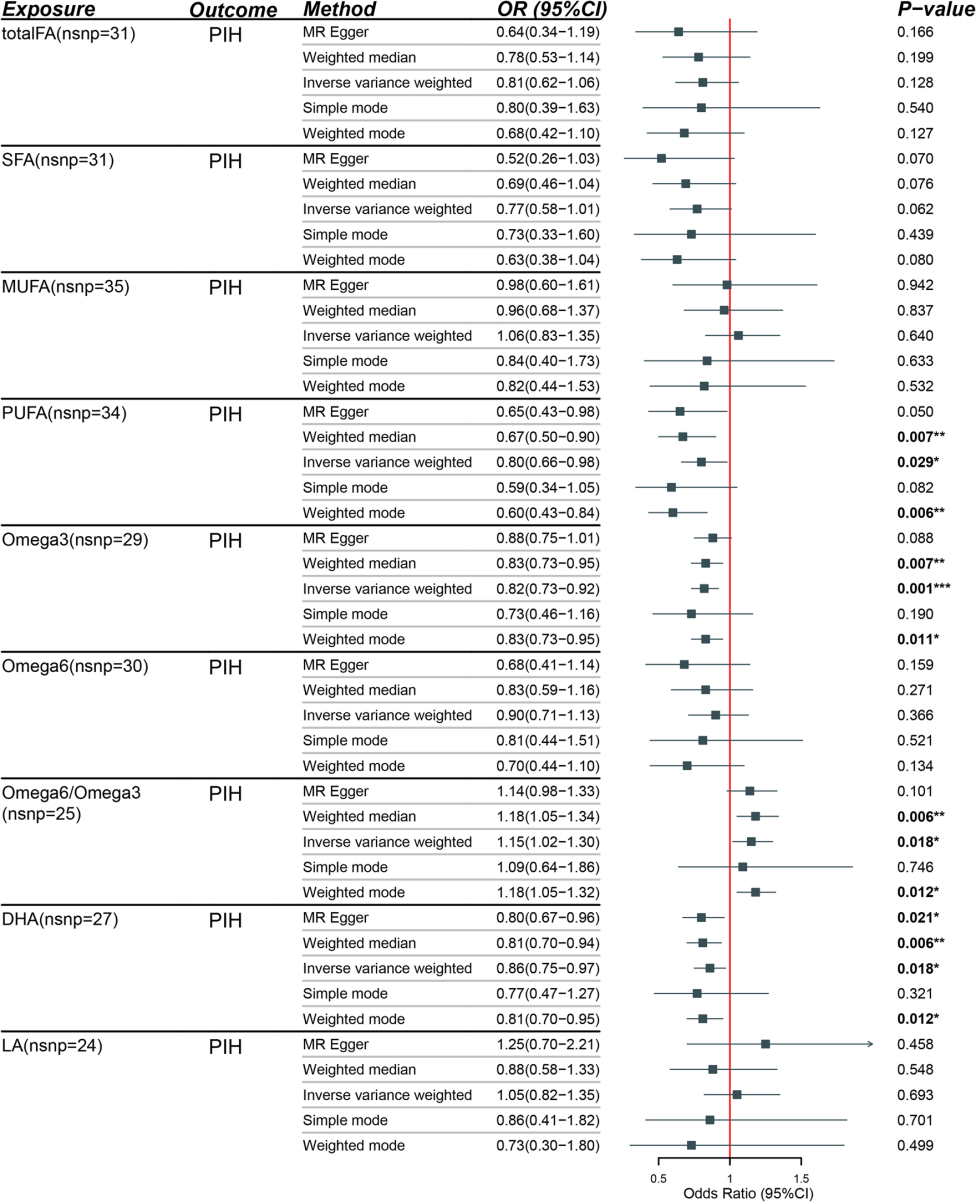
Results of univariable MR analysis of multiple fatty acids (FAs) and Pregnancy-Induced Hypertension (PIH)

Attention: In the graphs and tables, significance is denoted as follows: * for *P* < 0.05, ** for *P* < 0.01, and *** for *P* < 0.001.

According to the IVW method, one standard deviation (SD) increase in genetically determined Omega-3FA, PUFA, and DHA levels would respectively decrease the risk of PIH by 20%, 22%, and 16%. Conversely, a one SD increase in the Omega6/Omega3 ratio would increase the risk of PIH by 14%.

### Supplementary methods and sensitivity analysis

Beyond the primary IVW methods, additional statistical techniques such as MR-Egger, Weighted Median, Simple Mode, and Weighted Mode were employed to verify the accuracy of the main results relating to PIH. These methodologies continued to show no causal link between various types of Fatty Acids (Total FA, SFA, MUFA, Omega-6FA, LA) and PIH, as indicated by *P-*values  > 0.05. However, for PUFA, Omega-3, Omega-6/Omega-3 ratio, and DHA, the presence of a causal relationship with PIH could be shown by at least two of these four methods. Furthermore, Odds Ratios (OR) for the same exposure across all methods were consistently either above or below 1, providing reliable indicators of whether the exposure constituted a risk or protective factor.

Scatter plots were utilized to visualize the effect size for each MR method, effectively illustrating the correlation between exposure and outcome, as depicted in Supplementary Materials. Forest plots were applied to display individual SNP outcome estimates, while Funnel plots were used to represent the distribution of individual SNP effects. The leave-one-out analysis shows the impact on the outcome upon removing each effect, as shown in Supplementary Materials.

The scatter plot reveals that TFA, SFA, PUFA, Omega-3, Omega-6, and DHA are all likely to decrease the risk of PIH during pregnancy with track hypertension, while the Omega-6/Omega-3 ratio might pose a risk factor for PIH. The leave-one-out analysis indicates that SNP rs174564 exerts a substantial directional effect on Omega-3, DHA, and PUFA. Upon the removal of rs174564, the results turned negative, suggesting that this locus should be approached cautiously as it may be an essential gene for the protective factor of n-3 fatty acids against PIH.

In the sensitivity analysis, both heterogeneity and pleiotropy were evaluated separately, with the results presented in Table 3. (i) For heterogeneity detection, each Fatty Acid (FA) demonstrated a *P*-value exceeding 0.05 using both MR-Egger and IVW methods, indicating no statistical difference. This implies that the analysis of each FA was not subject to the influence of heterogeneity. (ii) For pleiotropy assessment, the *P*-values for each FA were also above 0.05, suggesting no statistical difference and implying that the analysis of each FA was not swayed by pleiotropy, thus not contravening Hypothesis 3. Consequently, the sensitivity analysis confirmed the robustness of the MR analysis results, unaffected by either heterogeneity or pleiotropy.

**Table 3:**
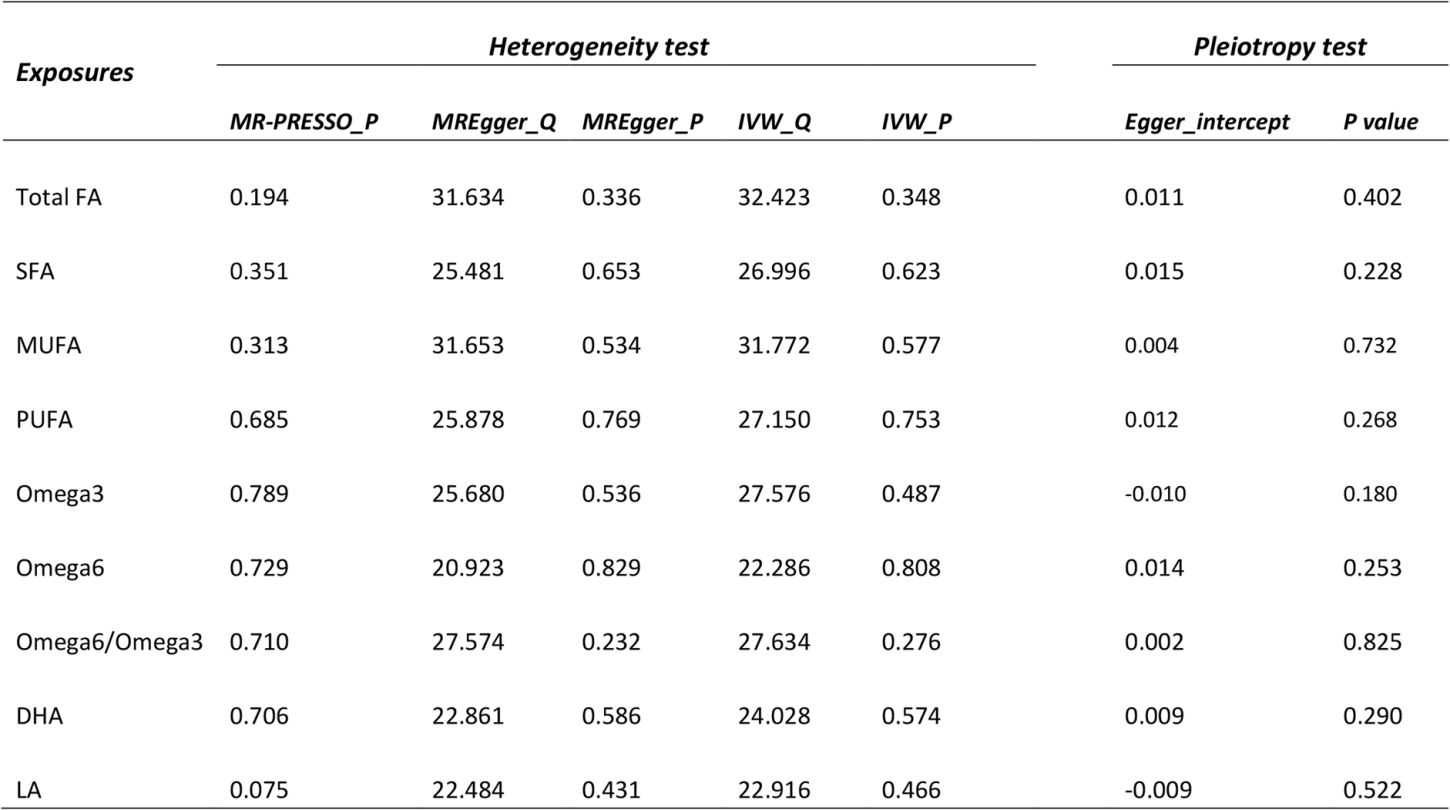
Results of heterogeneity and pleiotropy testing

### Multivariable MR

In the multivariable analysis, the Inverse Variance Weighted (IVW), MR-Lasso, and Weighted median methods were primarily utilized to assess the effects of correction in the confounding factors. Supplementary Material 1 presents the comprehensive estimated values and confidence intervals for the five models included in the multivariable analysis. All five models yielded MR Egger results  > 0.05, signifying model stability and the absence of pleiotropic effects. The direct influences of fatty acids on PIH were scrutinized following different hierarchical orders, as presented in Tables 4 and 5. The results, after accounting for various types of fatty acids, were as follows: DHA in Model 1 [LASSO, OR (95% CI): 0.84(0.72–0.99), *P* = 0.03]; Omega-3 in Model 2 [LASSO, OR (95% CI): 0.82(0.71–0.82), *P* < 0.001]; PUFA in Model 3 [LASSO, OR (95% CI): 0.83(0.73–0.95), *P* = 0.01]; DHA in Model 4 [IVW, OR (95% CI): 0.86(0.74–0.99), *P* = 0.04]; Omega-3 in Model 5 [IVW, OR (95% CI): 0.85(0.74–0.99), *P* = 0.03]. These results suggest that increasing levels of DHA, Omega-3, and PUFA could potentially reduce the risk of PIH. While all the results were positive exposures in the univariable MR analysis, the multivariable MR provided a different perspective, asserting a causal relationship via either LASSO or IVW methodologies.

**Table 4:**
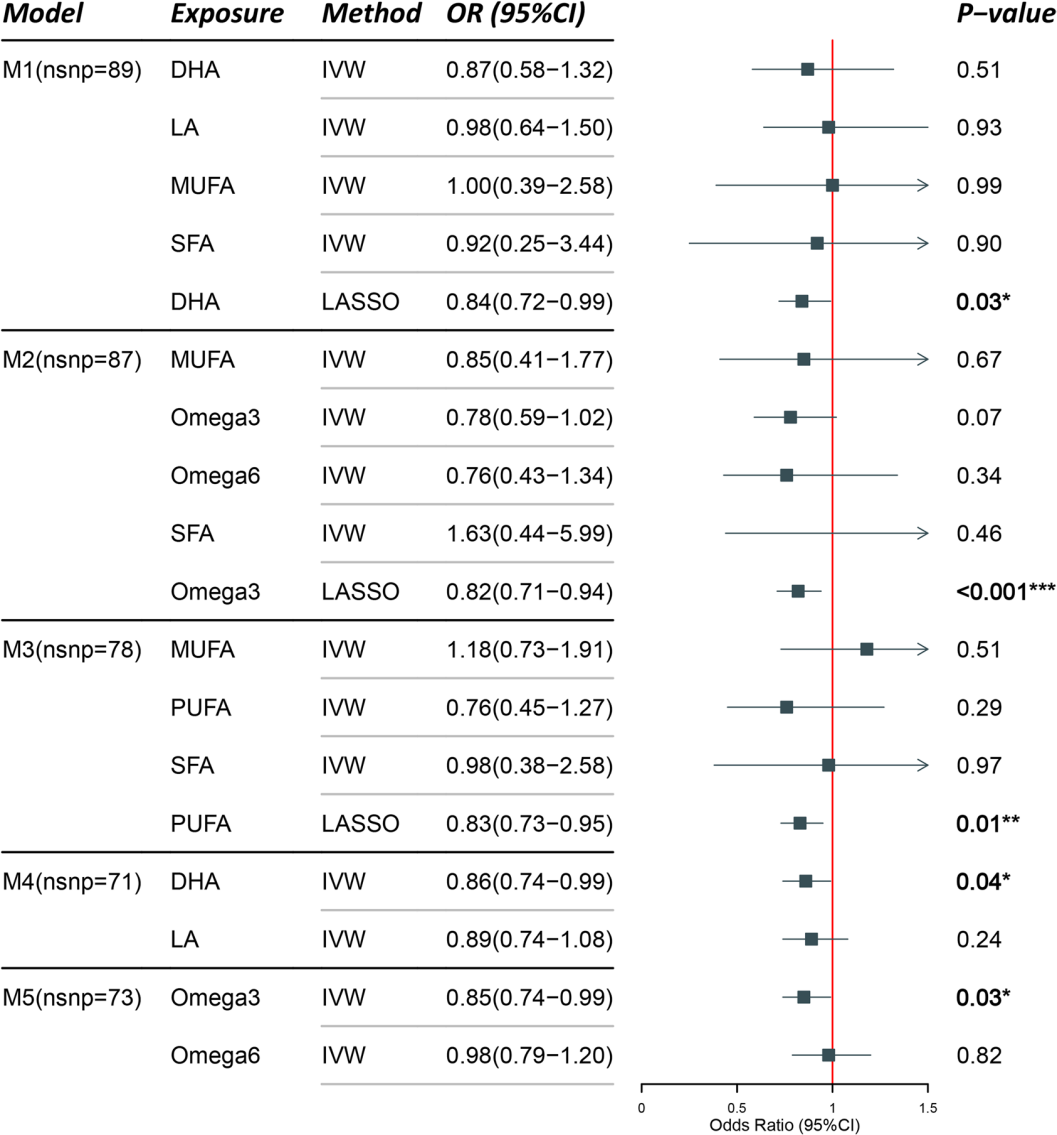
The multivariable MR results for each model. Model 1 considers DHA, LA, MUFA, SFA, and DHA. Model 2 includes MUFA, Omega-3, Omega-6, and SFA. Model 3 accounts for MUFA, PUFA, and SFA. Model 4 examines DHA and LA, while Model 5 considers Omega-3 and Omega-6

Attention: In the graphs and tables, significance is denoted as follows: * for *P* < 0.05, ** for *P* < 0.01, and *** for *P* < 0.001.

**Table 5:**
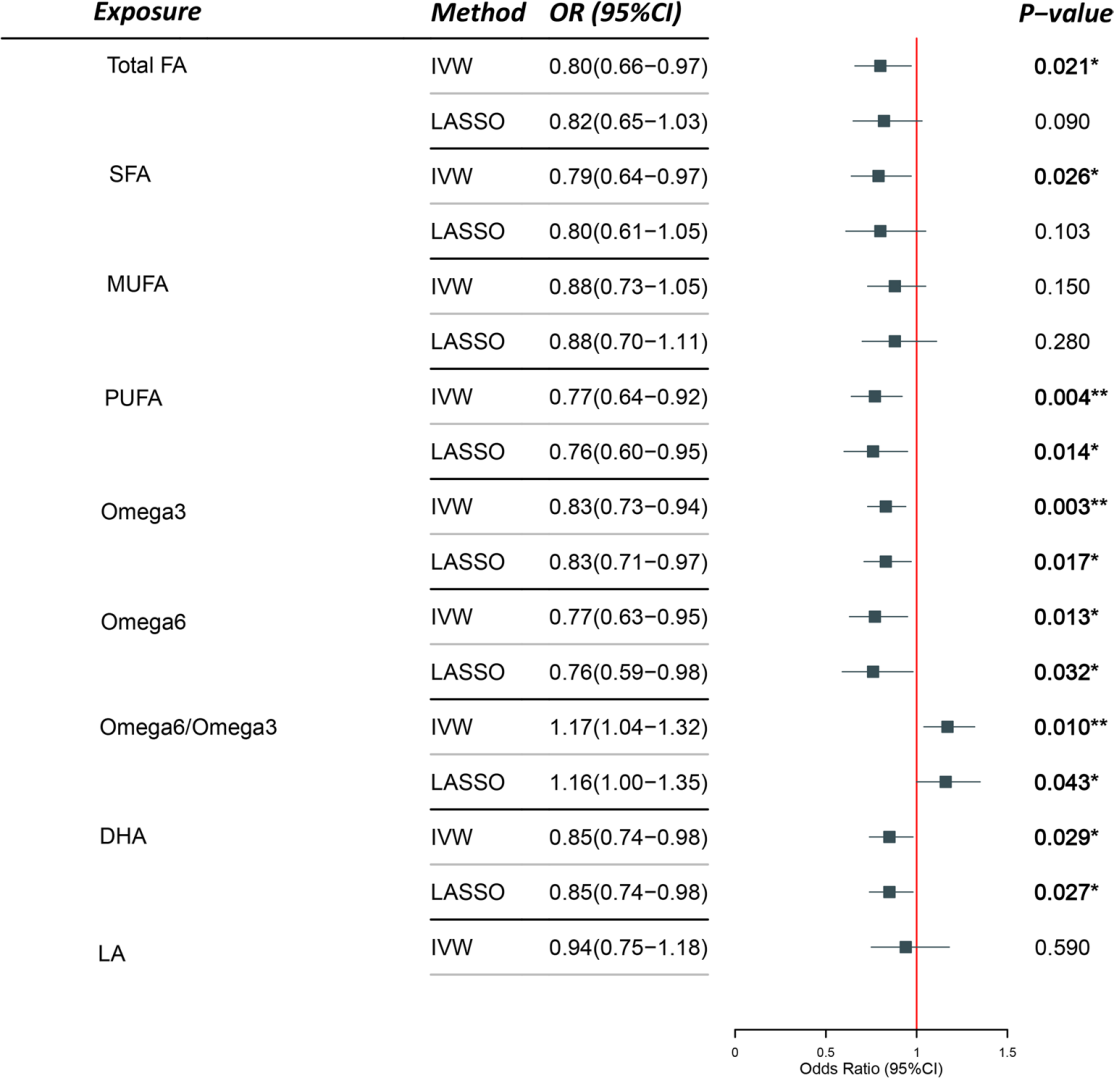
Results of multivariable MR analysis of balanced confounders BMI, diabetes mellitus, and hypertension

Attention: In the graphs and tables, significance is denoted as follows: * for < 0.05, ** for *P* < 0.01, and *** for *P* < 0.001.

According to the study, after adjusting for confounding factors, including BMI, diabetes, and high blood pressure, the following results were found:

All fatty acids, excluding SFA, MUFA, and LA, exhibited *P*-values > 0.05. Notably, SFA, MUFA, and LA exhibited at least one method indicating causality, despite *P*-values  > 0.05. Supplementary Material 1 illustrates each fatty acid’s estimated values and confidence intervals in the multivariable analysis, post confounder adjustment. The majority of the MR-Egger results’ *P*-values are < 0.05, which implies the existence of horizontal pleiotropy and the instability of the results. PUFA, Omega-3, Omega-6/Omega-3 ratio, and DHA maintained positive results, aligning with the outcomes of the univariable and multivariable MR analysis discussed above. The *P*-values in both the Inverse Variance Weighted (IVW) method and MR-Lasso remained less than 0.05, suggesting a potential causal relationship. However, a solid causal relationship is acknowledged for Omega-3 fatty acids [IVW, OR (95% CI): 0.83(0.73–0.94), *P* = 0.003] according to the Bonferroni method, given that the *P*-value is  < 0.005. These findings necessitate further verification for reliability, thereby warranting cautious interpretation and discussion.

## Discussion

In this study, MR was used to examine whether PUFAs are associated with PIH. The results revealed: (1) PUFA, Omega3, and DHA exhibit a causal relationship with PIH, suggesting that elevated concentrations of these fatty acids may decrease PIH risk; (2) The Omega6/Omega3 ratio also displays a causal relationship with PIH, indicating that higher ratios could contribute to PIH; (3) Specific single nucleotide polymorphisms (SNPs), particularly rs174564, were identified as potent influencers of these causal relationships; (4) However, our study could not conclusively establish a causal relationship between total fatty acids, SFAs, and MUFAs with PIH.

The exact pathogenesis of gestational hypertension and pre-eclampsia remains obscure. Identified factors include: (1) Vascular function abnormalities: in a healthy pregnancy, uterine blood vessels dilate to facilitate increased blood flow to the fetus [26, 27]. In gestational hypertension and pre-eclampsia, improper dilation of these blood vessels can lead to elevated blood pressure; (2) Immune system response: alterations in the immune system during gestation may contribute to the development of both disorders. For instance, abnormal maternal immune responses to the fetus could result in inflammation and irregular vascular function [28]. Our MR analysis affirmed this information, which identified the rs174564 gene as a crucial driver of Omega3 fatty acids, which serve as a protective factor against PIH.

The mechanism underlying the protective effect of omega-3 fatty acids on PIH risk remains unknown. Omega-3 fatty acids can be obtained from fish oil supplementation, consumption of omega-3-rich fish, or fortified foods. Past studies have associated Omega3 fatty acids with various biological roles including anti-inflammatory, anti-hypertensive functions, proteinuria reduction in nephropathy, and cardiovascular protection [29–32]. It is proposed that omega-3 fatty acids might protect uterine blood vessels via anti-inflammatory effects, thus diminishing the onset of gestational hypertension and mitigating existing conditions. Previous studies have equated the inhibitory impact of Omega3 fatty acids to the action mechanism of aspirin, recommended by ACOG guidelines for gestational hypertension and pre-eclampsia risk reduction [33].

The rs174564 gene, near the most influential genetic tool intron SNP in FADS2, plays a significant role in ω3 fatty acid production. FADS2, a desaturase protein, participates in the initial desaturation step of α-linolenic acid from C18:3 to C18:4. Recent MR studies demonstrate that upregulation of rs176564 gene expression reduces inflammatory responses [34, 35], albeit with varied effects across different tissues. Polyunsaturated fatty acid supplementation increases FADS gene activity in pregnant women [36], promoting anti-inflammatory and anti-thrombotic effects [37]. Due to limited evidence on these mechanisms, further experimental and clinical investigations are warranted.

At the genetic level, the discovery of the rs174564 gene as a substantial determinant in these causal relationships initiates possibilities for potential genetic testing that may facilitate the identification of individuals predisposed to PIH due to their genetic constitution. Future validation of these findings could lead to gene testing in pinpointing women potentially at elevated risk of PIH due to the rs174564 gene, thereby paving the way for customized dietary advice and intervention strategies.

In clinical management, the revelation that Omega-3 fatty acids, PUFAs, and DHA might confer protection against PIH has significant clinical ramifications, highlighting the vital role of nutrition in PIH management. This evidence could prompt healthcare providers to advise an augmented intake of these fatty acids through diet or supplements during prenatal care. Furthermore, this knowledge could aid in devising dietary guidelines for pregnant women to minimize PIH risks. Concurrently, the study suggests that an escalated Omega-6/Omega-3 ratio might increase PIH risk, hinting at a potential correlation between types of dietary fats and PIH vulnerability. Such understanding can substantially shape prenatal dietary guidance, emphasizing patients’ need to balance Omega-6 and Omega-3 fatty acids intake. This could mean reducing Omega-6-rich foods like certain vegetable oils and processed goods or elevating Omega-3-rich food consumption. Additionally, the absence of causal relationships between total fatty acids, MUFA, SFA, and PIH allows clinicians to focus dietary advice on fats specifically related to PIH, particularly Omega-3 and Omega-6 fatty acids.

It’s important to note that while these findings provide important insights and potential countermeasures for managing the risk of PIH, they should be interpreted with caution as they are based on an MR study. Further research, particularly randomized controlled trials, must confirm these findings before they can be applied in clinical practice.

## Strengths and limitations

Notable strengths of our study include substantial sample size and the application of both two-sample and multivariable MR analyses, significantly mitigating confounding bias and facilitating large-scale genetic data utilization on gestational hypertension. The reliability of the findings was confirmed using multiple MR analyses. Potential limitations of original RCTs or observational studies, such as fatty acid intake method and standardization challenges like dose, initiation time, and intake duration, are minimized in our MR analysis.

However, our study also presents limitations. Primarily, our results are based mainly on European ancestry data to mitigate racial effects, which restricts applicability to non-European populations. Also, the current dataset on PIH is relatively small and does not permit a multi-database test. Additionally, there might be some degree of sample overlap, as both exposure and outcome datasets are from European populations. Regrettably, assessing overlapping sample sizes remains challenging. Lastly, the PIH dataset includes data from people with gestational hypertension and pre-eclampsia. The causal relationship inferred between fatty acids and PIH in this study needs to be revisited regarding the dominating factors in these conditions. Therefore, it’s essential to further explore and validate this causal inference through RCTs with high certainty and rigorous control to confirm cause-effect existence.

## Conclusion

In summary, our MR study uncovered a protective role of Omega-3 fatty acids, DHA, and PUFAs against PIH. Conversely, a higher Omega6/Omega3 ratio was associated with an increased risk. However, our findings did not establish a causal relationship between Total fatty acids, MUFA, and SFA with PIH. Notably, the dominant factor typically emerges as a risk determinant for diminished pre-eclampsia. Future research must further scrutinize the causal link between fatty acids and gestational hypertension. This necessitates a substantial volume of meticulously controlled RCTs. To minimize potential bias, these trials should exercise stringent control over variables such as quantity, method, and timing of fatty acid intake.

### Supplementary Information

**Additional file 1.** 

## Data Availability

All data used in the present study were obtained from genome-wide association study summary statistics, which were publicly released by genetic consortia. All data is obtained from OpenGWAS (https://gwas.mrcieu.ac.uk/).

## Abbreviations

MR: Mendelian randomization
GWAS: Genome-wide association studies
PIH: Pregnancy-induced hypertension
BMI: Body mass index
SNPs: Single-nucleotide polymorphisms
RCTs: Randomized controlled trials
LD: Linkage disequilibrium
MUFA: Monounsaturated fatty acids
SFA: Saturated fatty acids
PUFA: Polyunsaturated fatty acid
LA: Linoleic acid
DHA: Docosahexaenoic acid
