# Supplementary material for: Fatty acids and pregnancy-induced hypertension: a Mendelian randomization study
Source: Lipids Health Dis. 2023 Aug 16;22:131. doi: 10.1186/s12944-023-01889-x (PMC10428562; doi:10.1186/s12944-023-01889-x)
Supplement: Supplementary file 1 — Additional file 1. [file 12944_2023_1889_MOESM1_ESM.zip › fig sup.docx]

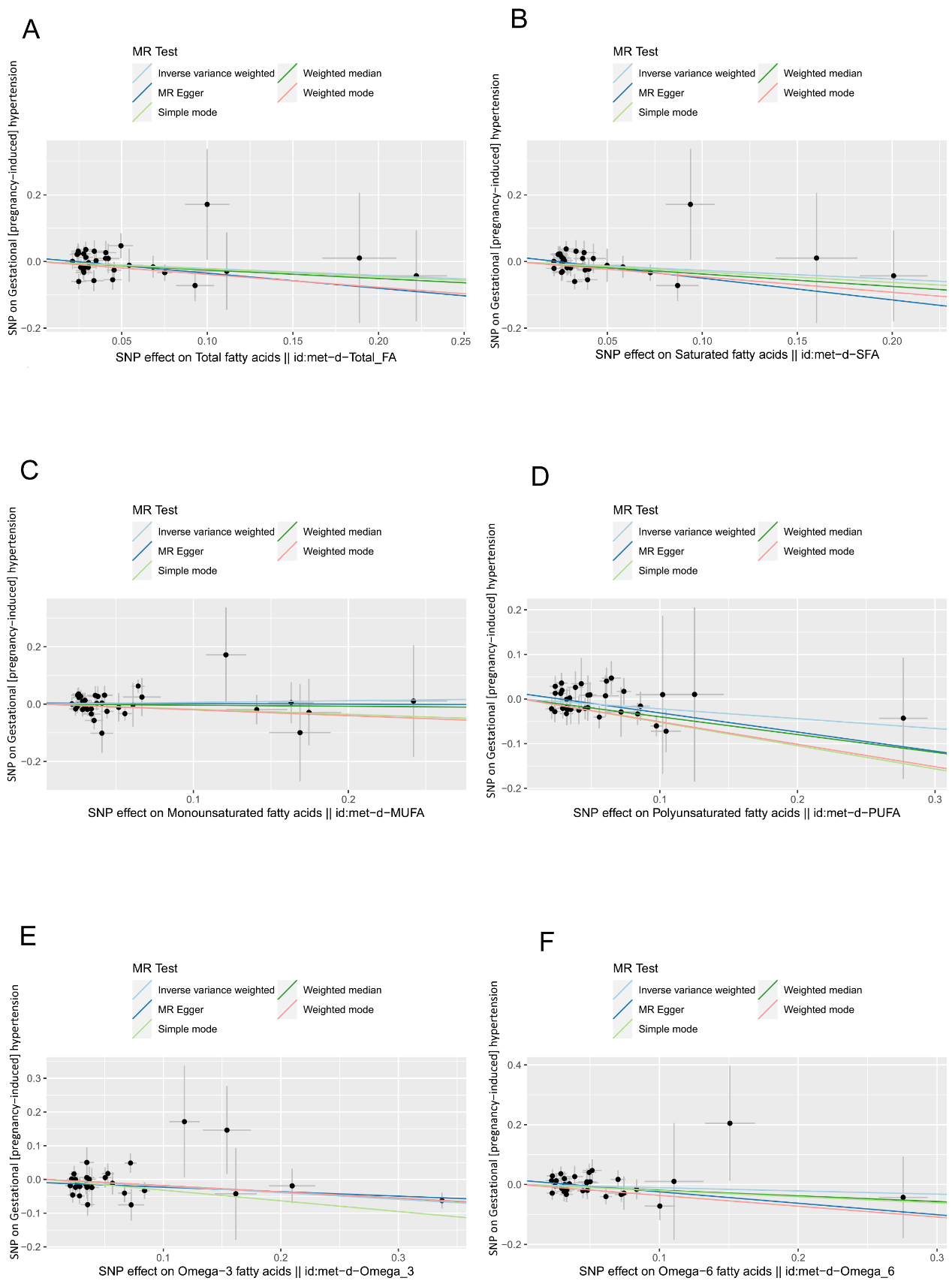


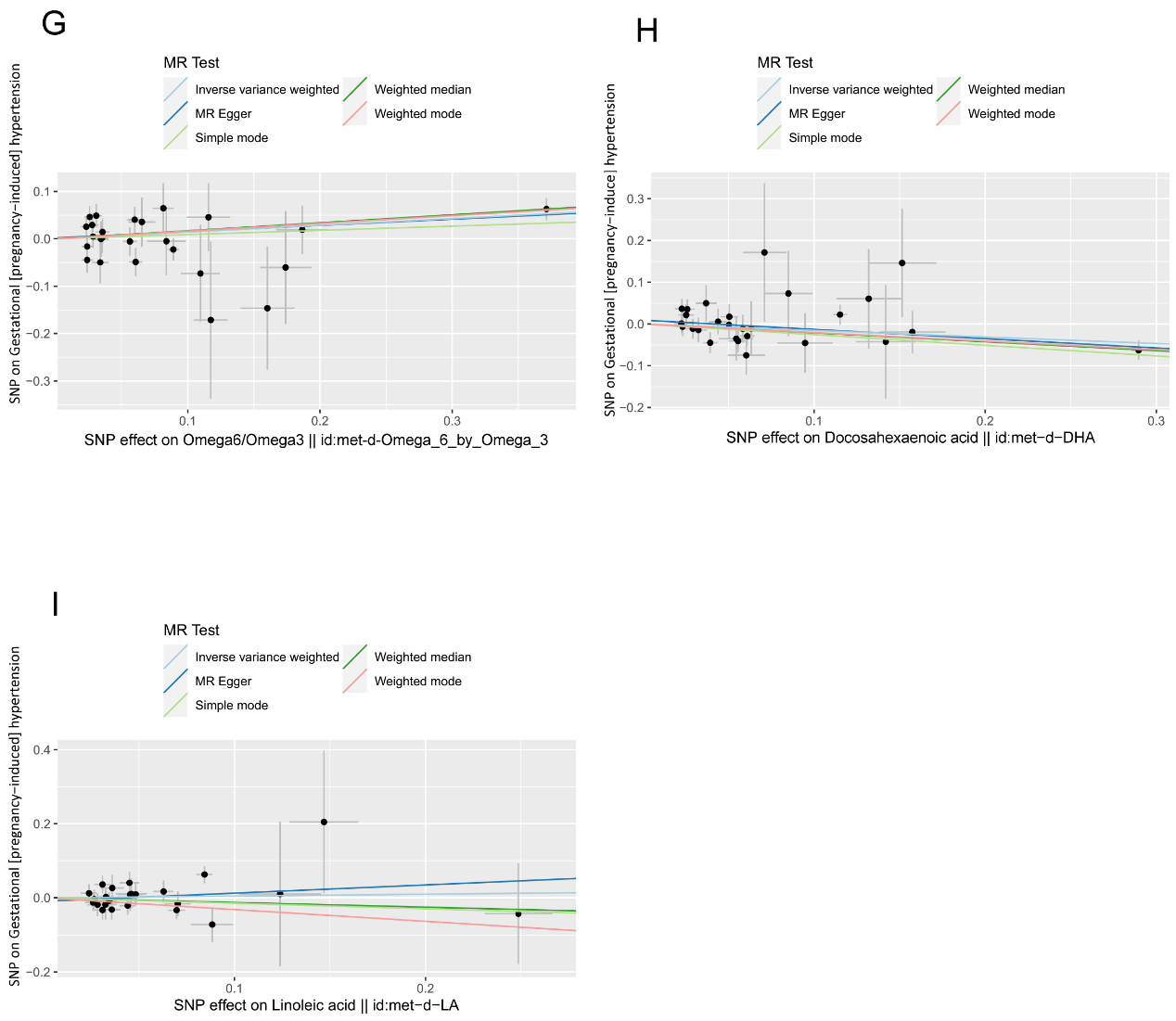


**Fig 1.** This scatter plot shows the relationship between fatty acids (FAs) and pregnancy-induced hypertension (PIH). On the X-axis, the effect of single nucleotide polymorphisms (SNPs), and standard errors (SEs) on each SNP was selected from the FA genome-wide summary association study (GWAS). On the Y-axis are the SNP effects and SEs in PIH genotype-wide association studies (GWAS). (A) Analysis of Total FA and PIH;(B) saturated FA and PIH;(C) monounsaturated FA and PIH;(D) polyunsaturated FA and PIH;(E) Omega-3 FA and PIH;(F) Omega-6FA and PIH;(G) Omega-6-6 FA / Omega-3 FA and PIH;(H) DHA and PIH;(I) LA and PIH.


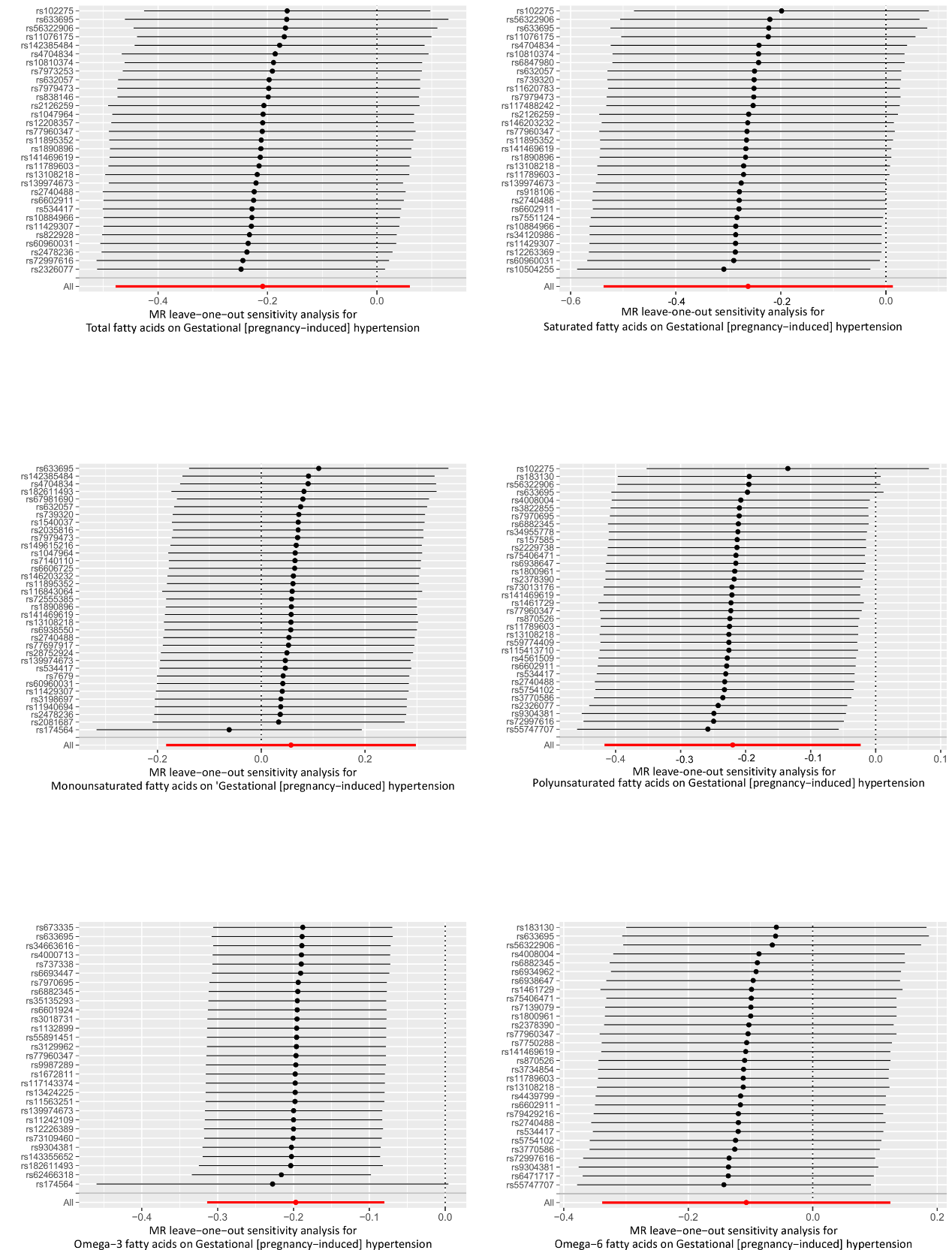


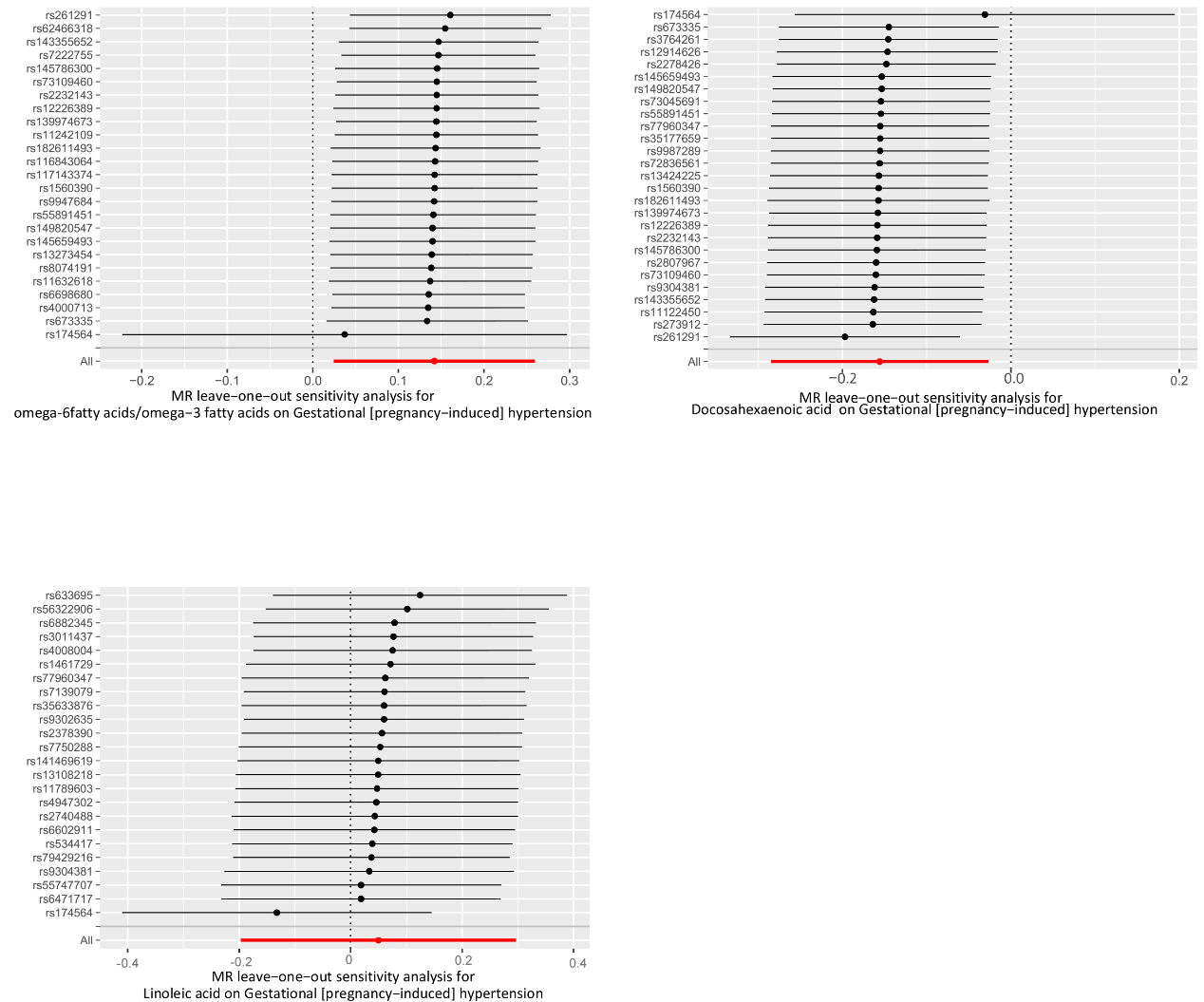


**Fig2.** Leave-one-out method result chart. (A) Total FA SNPs Analysis; (B) Saturated FA SNPs Analysis; (C) Monounsaturated FA SNPs Analysis; (D) Polyunsaturated FA SNPs Analysis; (E) Omega-3 FA SNPs Analysis; (F) Omega-6 FA SNPs Analysis; (G) Omega-6 FA /Omega-3 FA SNPs Analysis; (H) DHA SNPs Analysis; and (I) LA SNPs Analysis.
